# A New Treatment of Smallpox

**Published:** 1880-07

**Authors:** 


A New Treatment of Smallpox.—A Lyons doctor has discovered a new mode of treatment in smallpox which he asserts is pre-eminently curative. As it is well known that death arrives in this disease at two distinct periods—in the first three days when the eruption with difficulty manifests itself, or when the multitude of pustules inflame and throw into the circulation the suppuration of the face, chest, and extremities successively—it is against this last danger that the doctor in question has adopted this treatment, which consists in painting toward the end of the seventh day, when the suppurating fever commences, the whole surface of the body, commencing at the feet, with a mixture of glycerin three parts, tincture of iodine one part. This painting process is renewed every four hours until the twelfth day of the disease. Our *confrere* asserts that he treated thus seven cases of confluent smallpox successively.

